# Factors That Motivate Provider Switching: The Patients' Perspective

**DOI:** 10.1111/1475-6773.70028

**Published:** 2025-08-14

**Authors:** Onyi Dillibe, Rahul Singh, Norman A. Johnson

**Affiliations:** ^1^ Department of Information Systems & Supply Chain Management University of North Carolina at Greensboro Greensboro North Carolina USA; ^2^ Department of Decision and Information Sciences University of Houston Houston Texas USA

**Keywords:** critical incident analysis, patient switching, qualitative research

## Abstract

**Objective:**

To generate evidence regarding the specific critical incidents that prompt patients to switch care providers.

**Study Setting and Design:**

Building on existing work on customer switching behavior, we applied the critical incident technique (CIT) to the health services research context and analyzed primary data obtained from 555 US‐based patients who reported switching providers between 2018 and 2022 to develop a typology of the critical incidents that prompt patients to switch healthcare providers.

**Data Sources and Analytic Sample:**

Data were obtained from an online survey of adult US‐based patients who reported switching primary care providers (PCPs) for non‐insurance‐related reasons. The survey was conducted from August to September 2022 using a quota sampling approach.

**Principal Findings:**

We found eight critical incident categories associated with patient switching: service encounter failures, pricing, competitor attraction, inconvenience, core service failures, involuntary switching, breakdown in shared decision‐making, and service environment perception.

**Conclusion:**

We offer explanations and suggest potentially useful evidence‐based strategies for further investigation.

**Summary**

- What is known about this topic
  ○ Patient switching has far‐reaching consequences for healthcare systems. These include disruptions in care continuity that may result in poor health outcomes, increased healthcare costs, loss of revenue, and loss of reputation.
  ○ Health systems and providers acknowledge that they need a better understanding of why patients leave one provider in favor of another.
  ○ While studies have investigated factors that prompt switching behavior, research from the patient's perspective that prompts such switching behavior is lacking.
- What this study adds
  ○ While most patients switch providers for involuntary reasons (e.g., relocation), a significant share of patients actively switch in response to dissatisfactory service encounters, underscoring patient agency in driving quality improvements.
  ○ Breakdowns in shared decision‐making and perceptions of the service environment emerged as two key previously unrecognized drivers of voluntary patient switching.
  ○ These two factors independently motivated switching, beyond traditional factors such as cost or convenience.

## 1 Introduction

Patient switching behavior occurs when patients leave their current healthcare providers for services offered by other providers [1]. Patients are motivated to switch healthcare providers for various reasons, including the unavailability of required services, dissatisfaction with service encounter aspects [2, 3], or personal preferences [4, 5]. Switching providers poses challenges for patients, providers, and healthcare stakeholders. For patients, switching can disrupt care continuity, resulting in poorer health outcomes, increased hospital admissions, and higher mortality [6, 7]. Since trust with providers is built over time, switching also disrupts trust development in patient‐provider relationships, which contributes to patients' reluctance to share relevant health information and increased dissatisfaction with new providers [8]. For providers, patient switching impedes understanding the patient's condition, increasing the likelihood of medical errors and higher costs of care [9]. Over time, these may contribute to lower job satisfaction and increased burnout for providers [10, 11]. For provider organizations, switching can reduce revenues by as much as 10% [12] and damage reputation as prospective patients view patient switching as indicative of poor care quality [13]. This perception can spiral into market share declines and threaten the provider's long‐term viability [14]. Patient switching can raise care delivery costs, driving up overall expenses and insurance premiums due to information fragmentation [9].

Despite these demerits, switching providers can also benefit patients and healthcare markets immensely. For patients, switching providers is an important mechanism for exercising choice and influencing the quality of care they receive. It empowers patients to redirect service experiences by moving services toward providers who they perceive offer services that are of better quality, more affordable, or better aligned with patients' values [15, 16]. At market levels, patients' ability to change providers is key for system‐wide improvement in quality, costs, and availability of care through two complementary mechanisms. First, when patients switch in response to quality deficiencies such as medical errors or poor interpersonal experiences, they redistribute volume from lower‐performing providers toward those offering superior services. Second, the possibility that patients may switch providers can be a powerful incentive for providers to improve service delivery standards and pay more attention to patient needs or prices [17, 18]. Over time, these dynamics can result in systemic improvements in healthcare. Thus, for a myriad of reasons, it is critical to understand why patients switch providers—to mitigate undesirable consequences and identify opportunities for system‐wide improvements in the quality of care patients receive.

Although some research has investigated factors associated with patient switching behavior, most have focused on system and organizational‐level structural factors, or provider perspectives, often using secondary data—such as claims—which show switching behavior but lack the nuance and context to explain why switching occurred [14, 19, 20, 21, 22, 23, 24]. Few studies have examined switching behavior from the patient's perspective [21, 25]. These are limited by their reliance on factors determined a priori, which may overlook motivations germane to healthcare or to patients' experiences. Some studies use composite measures of service quality, which obscure specific aspects of the patient experience that result in switching behavior [21, 25]. As a result, there is a limited understanding of how patients perceive and interpret the factors that prompt them to switch providers. Without patient input, interventions and policies derived from organizational or provider‐centric studies may overlook the actual reasons for switching, potentially rendering them ineffective.

In a seminal study, Keaveney [26] identified eight critical incident categories to explain customer switching behavior, from the customer's perspective. However, whether these findings apply to healthcare remains uncertain, given its unique attributes. First, healthcare services are highly specialized, characterized by information asymmetry (patients often know less than providers), and care benefits unfold gradually. Therefore, it is difficult for patients to accurately evaluate the technical quality of their care [27, 28]. Consequently, patients rely on the observable and non‐technical aspects to assess care quality [29]. Therefore, relational aspects of service encounters can have outsized impacts on patient switching behavior. Second, unlike most service markets, patients are often insulated from healthcare costs by insurance and other third‐party payers. Therefore, patients may be less sensitive to prices than consumers in other industries. Third, patients facing difficult diagnoses and health‐related stress often experience heightened negative emotions such as fear and anxiety [29], These emotions can make patients rely more on providers for extra support. Such emotional states shape patients' perceptions of their experiences, influencing switching decisions differently from other consumers. Drawing on Keaveney's [26] model of customer switching in service industries, this study aims to identify factors that motivate patients to switch care providers, from the patient's perspective. We extend Keaveney's [26] definition of critical incidents into healthcare and define a *critical incident as any event or combination of events between patients and providers where patient switching was the outcome*. We use the critical incident technique [30] (CIT) to analyze primary data from a survey of U.S. patients who reported switching providers.

## 2 Methods

### 2.1 The Critical Incident Technique

The critical incident technique (CIT) is a systematic, inductive grouping procedure that relies on first‐hand reports from subjects (actual patients) and identifies groups of grounded events associated with outcomes (patient switching behavior) [31, 32]. These *critical incidents* (CI) are complete enough to draw inferences about respondents' motivations. Incidents are elicited by posing a series of open‐ended questions, and subjects have complete freedom in responding. Thus, CIT makes no a priori determination of importance and does not force respondents into predetermined frameworks. CIT provides rich data to understand the incident from the respondent's perspective, while accounting for cognitive, affective, and behavioral elements [32]. Incidents are deemed critical when they significantly contribute to the outcome of interest [30, 33].

Prior studies show that CIT reliably identifies stable categories across respondents that are valid and relevant regarding the content identified, and are important antecedents to the phenomenon of interest [31, 34]. Therefore, we chose CIT as a reasonable choice to capture patients' perspectives on switching behavior.

### 2.2 Data Collection

We conducted an online survey between August and September 2022 of US‐based adults (age ≥ 18 years) who reported switching primary care providers (PCPs) for non‐insurance‐related reasons within the past 5 years. The 5‐year time frame considers the infrequent utilization of primary healthcare services and non‐emergency healthcare services during and immediately following the COVID‐19 pandemic. Our time frame conforms to widely accepted CIT data collection recommendations and allows sufficient time to observe relevant specific behaviors [31].

We used Qualtrics Research Services to identify and recruit participants, disseminate the questionnaire, and collect data. Qualtrics leverages its access to actively managed pools of diverse individuals who agree to be contacted for research studies [35] to identify potential participants likely to meet our inclusion criteria. They were emailed a URL to the questionnaire. Responses were solicited from 2000+ participants to achieve a target sample size of approximately 650 complete responses using a quota‐based sampling approach designed to approximate national diversity based on key demographic distributions (e.g., gender, geographic region, race/ethnicity, etc.) among U.S. adults. This approach was selected because a well‐defined sampling frame of patients who switched providers is not known, making probability‐based alternatives infeasible. Qualtrics paid a small amount ($2–$4) to qualified participants who met the inclusion criteria and completed the survey. We obtained 670 responses with a response rate of approximately 30%. Of these, 115 responses were excluded because responses were incomplete or unrelated to the questions, and 555 responses remained in our analytic sample. Table 1 presents the descriptive statistics of our analytical sample in comparison to the full quota‐balanced sample. The table indicates no statistically significant difference between both samples across all demographic categories.

**TABLE 1:** Sample description.

Metric	*N*, 555	(%), (100%)	*N*, 670	(%), (100%)	∆	*p*
Age						
18–24	119	21.44%	146	21.79%	−0.35%	0.88
25–44	127	22.88%	171	25.52%	−2.64%	0.28
45–64	144	25.95%	177	26.42%	−0.47%	0.85
65+	165	29.73%	176	26.27%	3.46%	0.18
Gender						
Female	292	52.61%	350	52.24%	0.37%	0.90
Male	261	47.03%	318	47.46%	−0.43%	0.88
Non‐Binary	2	0.36%	2	0.30%	0.06%	0.86
Marital status						
Divorced	72	12.97%	81	12.09%	0.88%	0.64
Married	237	42.70%	286	42.69%	0.01%	1.00
Separated	10	1.80%	13	1.94%	−0.14%	0.86
Single	193	34.77%	244	36.42%	−1.65%	0.55
Widowed	43	7.75%	46	6.87%	0.88%	0.55
Household income						
< $25,000	59	10.63%	72	10.75%	−0.12%	0.95
$25,000–$49,999	156	28.11%	195	29.10%	−0.99%	0.70
$50,000–$99,999	208	37.48%	252	37.61%	−0.13%	0.96
$100,000–$199,999	109	19.64%	124	18.51%	1.13%	0.62
$200,000+	23	4.14%	27	4.03%	0.11%	0.92
Education						
Some high school or less	15	2.70%	18	2.69%	0.01%	0.99
High school diploma or GED	321	57.84%	388	57.91%	−0.07%	0.98
Bachelor's degree	172	30.99%	210	31.34%	−0.35%	0.89
Master's degree or above	47	8.47%	54	8.06%	0.41%	0.80
Race/Ethnicity^a^						
American Indian/Native American or Alaska Native	10	1.80%	10	1.49%	0.31%	0.68
Asian	30	5.41%	39	5.82%	−0.41%	0.76
Black or African American	60	10.81%	78	11.64%	−0.83%	0.65
Native Hawaiian or Other Pacific Islander	1	0.18%	1	0.15%	0.03%	0.90
Other	19	3.42%	20	2.99%	0.43%	0.67
White or Caucasian	435	78.38%	522	77.91%	0.47%	0.84
Employment						
Disabled, not able to work	29	5.23%	31	4.63%	0.60%	0.63
Employed, working 1–39 h per week	154	27.75%	199	29.70%	−1.95%	0.45
Employed, working 40 or more hours per week	152	27.39%	201	30.00%	−2.61%	0.31
Not employed, NOT looking for work	17	3.06%	19	2.84%	0.22%	0.82
Not employed, looking for work	36	6.49%	44	6.57%	−0.08%	0.96
Retired	167	30.09%	176	26.27%	3.82%	0.14
Healthcare utilization						
Less than once a year	61	10.99%	75	11.19%	−0.20%	0.91
Every 7 to 12 months	136	24.50%	156	23.28%	1.22%	0.62
Every 3 to 6 months	247	44.50%	292	43.58%	0.92%	0.75
Every 1 to 2 months	81	14.59%	105	15.67%	−1.08%	0.60
More than once every month	30	5.41%	42	6.27%	−0.86%	0.52
Transportation challenges						
Never	354	63.78%	414	61.79%	1.99%	0.47
Seldom	86	15.50%	100	14.93%	0.57%	0.78
Sometimes	78	14.05%	105	15.67%	−1.62%	0.43
Often	28	5.05%	39	5.82%	−0.77%	0.55
Almost always	9	1.62%	12	1.79%	−0.17%	0.82
Number of chronic conditions						
None	191	34.41%	244	36.42%	−2.01%	0.46
One	127	22.88%	158	23.58%	−0.70%	0.77
Two	121	21.80%	146	21.79%	0.01%	1.00
Three or more	116	20.90%	122	18.21%	2.69%	0.24
Location						
Rural	131	23.60%	153	22.84%	0.76%	0.75
Sub‐Urban	272	49.01%	334	49.85%	−0.84%	0.77
Urban	152	27.39%	183	27.31%	0.08%	0.98

^a^ The Race/ethnicity classification was self‐reported by study respondents, based on the US Office of Management and Budget's (OMB) Revisions to the Standards for the Classification of Federal Data on Race and Ethnicity, which specifies five minimum race/ethnicity categories for classifying data on race and ethnicity: American Indian/Native American or Alaskan Native, Asian, Black or African American, Native Hawaiian or Other Pacific Islander, and White (OMB, 1997). In addition, we included the category “Other” to capture the race/ethnicity of subjects who self‐reported that their race/ethnicity does not belong to any of the five categories listed above.

We asked respondents to “think about the last time that you switched care providers” and “Tell us, in your own words, why you switched from this care provider to a different one.” They described switching behavior details by responding to: “Tell us exactly what happened: how long ago this happened, where you were, what the care provider or their staff did or failed to do, what you said, how you felt, etc.”; “how long had you been with the provider you switched from.” We collected respondents' demographic information. We pretested the survey using a pilot study with 43 respondents and addressed survey flow and verbiage issues.

### 2.3 Analysis—Classification of Critical Incidents

We adopted a two‐stage approach to categorize our responses into critical incident categories (CICs). First, we provided two coders (A and B) definitions and examples of Keaveney's [26] eight CICs, including: core service failures, failed service encounters, pricing, inconvenience, response to service failures, competition, ethical problems, and involuntary switching. Coders distilled, categorized, and integrated response data into this framework and suggested new CICs when Keaveney's model did not adequately capture the patient experience, explaining switching behavior [33]. Incidents that did not fit the original framework were deemed healthcare specific. For consistency, the coders independently categorized all events from a random sample (10%) of data and resolved discrepancies in the CICs by discussion, before proceeding to code the larger dataset. Two new healthcare specific CICs were identified and included in our framework: *Breakdown in Shared Decision‐making* and *Perception of Service Environment*; and the two CICs from Keaveney's framework, which did not occur in our sample: *Employee Responses to Service Failures and Ethical Problems*, were excluded. Infrequently mentioned responses were classified as “other.” Based on Krippendorff's *α*, intercoder reliability between coders was 0.90, exceeding the 0.67–0.80 norm for good reliability [36, 37], indicating consistency between coders.

Then, three other coders (C, D, and E) systematically applied our refined coding frame to a random 10% sample of the dataset to assess CICs and resolve disagreements by discussion. Each coder then independently analyzed the remaining 90% of data. The final coding frame exhibited good intercoder reliability (Krippendorff's *α* = 0.74), a high agreement considering the coding frame comprised eight categories, including two new ones. Table 2 describes CICs and subcategories in our final refined coding frame.

**TABLE 2:** Definitions of critical incidents in the final coding frame.

Category	Definition	Subcategories
Core service failures	Critical incidents resulting from mistakes or other technical problems with the service.	Incorrect diagnoses Negligence Medical procedure Errors Prescription drug errors Administrative errors
Service encounter failures	Critical incidents due to negative evaluations of personal interactions between the patient and the service employee.	Lack of empathy Unprofessionalism Impoliteness Unresponsiveness Unknowledgeable Unarticulated affective judgment
Pricing	Critical switching behaviors where patients reported leaving their current provider because of price‐related concerns, such as price increases, unfair pricing practices, or patients' inability to afford services.	High prices Comparative price assessments Price increases Unaffordable pricing Unfair pricing practices
Inconvenience	Incidents where patients felt inconvenienced by the provider's location, hours of operation, wait times for service or to appointments.	Location/hours of operation Too long to schedule appointment Long wait for service delivery
Attraction by competitors	Critical incidents where patients perceived that superior service or value was available elsewhere, rather than negative experiences with the provider.	Better quality service Better benefits or value More highly skilled professionals Greater ease of accessing service
Involuntary switching	Critical incidents that were beyond the control of either the patient or the service provider.	Patient‐related circumstances Provider‐related Organizational issues
Breakdown in shared decision‐making	Critical incidents related to a disparity between patients' expectations or beliefs regarding their diagnosis and treatment and those of their providers	Disparities in range or volume of services expected Disparities in patient‐provider service philosophies Disparities in expected outcomes
Perception of the service environment	Critical incidents where patients switched because of dissatisfaction with the design of the service delivery process or physical environment.	Physical space Staff turnover Role assignment Biases toward provider or other customers

*Source:* Adapted from Reference [14].

## 3 Results

### 3.1 Critical Incident Categories

Table 3 summarizes the frequency of occurrence for each CIC, illustrated with respondents' representative quotes. While the majority of 555 respondents (492, 88.6% of all respondents) cited a single CIC to explain switching behavior, some (63, 11.4%) cited multiple. These were labeled Multiple Factor Incidents versus Single Factor Incidents, where only one CIC explained switching behavior. The 555 respondents in our study cited a total of 563 single and multiple factor incidents to explain switching. For example, Service Encounter Failures were cited 97 times: 59 times as a CIC and 38 times as one of multiple CICs explaining switching. Also, within a single CIC, some respondents cited multiple sub‐categories to explain switching behavior.

**TABLE 3:** Frequency of Critical Incident Category Occurrences for Patients Voluntarily Switching Providers, with Representative Comments.

Critical incident category	Voluntary switching incidents		
	*N*	%	Representative comments
Service encounter failures	97	30.2%	*I was rushed in and out. Sometimes she wouldn't even come into the room just stand at the door. I felt like an item she needed to check off her list*. *She didn't care about how I felt…When I would go to talk to her how I was feeling she would fall asleep*. *Was extremely rude and didn't listen to my concerns*.
Attraction by competitors	47	14.6%	*I felt the other provided more service and better*. *Better deals and advantages*. *Better doctors*.
Inconvenience	41	12.8%	*Doctor started scheduling appointments more than 4 months out*. *We couldn't get appointments, and when we did, we had to wait at least an hour before being seen*. *I moved to a provider local to my community*. *Transportation to previous establishment was too long*.
Pricing	40	12.5%	*Too expensive…I thought he was a hack*. *They upped their prices and I was no longer able to afford it*. *They were overcharging me*.
Core service failures	27	8.4%	*A nurse administered a vaccine I was contraindicated (allergy) for and wasn't supposed to have received based on CDC guidelines regardless of my allergy*. *Because I had complications from a procedure with that doctor*. *They dropped the ball and forgot to submit/approve the prior authorization for a prescription*.
Breakdown in shared decision‐making	23	7.2%	*Didn't like what the Dr was telling me to do regarding my arthritic knees* *I found an online site that described all my symptoms I was having. I have learned that most doctors are clueless that this happens and that antidepressants are giving out like candy instead of doing stuff through natural supplements*. *She didn't manage my healthcare like I think she should*.
Perception of service environment	19	5.9%	*The staff kept changing. I got tired of new people popping up when I went to doctor office*. *Changed to a more formal doctor's office as opposed to a clinic*. *After all the Covid stuff my doctor was still not seeing people in his office. So, it was time to change. This happened after most of the Covid problems were over and just didn't like how he wasn't seeing people in the office*.
Others	27	8.4%	

Involuntary CICs were the most frequently cited reasons for switching, accounting for 53.0% (*n* = 294) of all reported incidents. These incidents did not reflect patients' voluntary choice to switch; rather, they underscored systematic issues or factors beyond their control. On the other hand, voluntary CICs, which reflect patients' decisions to switch providers due to the nature of the care and service received, accounted for 48.5% (*n* = 269) of all incidents cited. Given the large proportion of involuntary incidents, we divided our analysis into two broad parts—involuntary and voluntary switching incidents.

We first discuss CI in the involuntary switching category, with proportions of all incidents in the category and sub‐category, with representative comments from respondents. Then, we discuss the CICs and related sub‐categories of voluntary switching and report proportions of CI from that specific category, along with representative comments for patients making a voluntary decision to switch providers.

#### 3.1.1 Involuntary Switching

Involuntary CICs, involving factors “beyond the control of either the customer or service provider,” [14] accounted for 53.0% of all incidents. Three subcategories of involuntary switching were identified: provider circumstances, patient circumstances, and organizational issues. Providers' circumstances, 46.9% of CI, included: the provider moved, retired, or passed away. 49.7% of involuntary switching related to patient circumstance changes, including the patient moving, adverse life events (e.g., job loss or death in family), or needing more specialized care. One patient noted: “I became very sick with several autoimmune disorders, and I almost died. I realized then that the advanced care that I needed didn't exist there.” The 3.4% remaining involuntary switching CI referred to organizational issues where insurance partnerships or the practice changed.

#### 3.1.2 Voluntary Switching

Voluntary switching CIs reflect patients' voluntary choice to switch care providers. The following report on these CIC includes *the proportion of all voluntary switching incidents* attributed to that CIC. We discuss the CIC and associated sub‐categories, with respondents' representative comments.

##### 3.1.2.1 Service Encounter Failures

In the service encounter failure CIC, patients attribute switching behavior to negative evaluations of interactions with care provider or other employees. These interpersonal aspects of healthcare service encounters are described as “affective care,” “expressive care,” or “bedside manners” [38] wherein customers evaluate interpersonal aspects of care along cognitive and affective dimensions. Cognitive dimensions involve patients' objective evaluations of positive and negative aspects of service employees' behavior and attitudes relative to patients' personal expectations or accepted standards. Affective dimensions are subjective evaluations of patients' emotions generated during patient–service employee interactions [39, 40]. Affective evaluations may occur without extensive cognitive encoding in memory, and some patients have difficulty verbalizing the origins of their emotional responses [40]. We describe these as “unarticulated affective judgment.”

Of all voluntary switching incidents, 30.2% of patients cited service encounter failures as one reason for switching providers. Of these, 16.2% switched because of unarticulated affective judgment (“didn't like the last one”). We also identified five other subcategories of service encounter failures incidents: (i) insufficient empathy (44.1%), where patients switched because providers were not adequately caring, unhelpful, or rushed through appointments not listening to patients' complaints (“Didn't feel as though my needs were being properly addressed or that I was being heard”); (ii) unprofessionalism (15.3%), where providers seemed disorganized, unreliable, or otherwise inadequately prepared for the appointment (“not well informed of my treatment plan”); (iii) impoliteness (12.6%), where respondents felt service providers were disrespectful (“*rude customer service*”); (iv) unresponsiveness (11.7%), where respondents reported delay or failure to communicate with patients (“provider was not following through on a particular procedure … I had to call over and over again to get her to and go over my results.”); and (v) lack of knowledge (6.3%), where patients perceived the provider was inexperienced or incompetent (“[Provider] was inexperienced…He did not listen to my heart, take my temperature, weight, blood pressure, etc. He mispronounced medications”).

##### 3.1.2.2 Attraction by Competitors

Attraction by Competitors (14.6% of all voluntary switching incidents), involved switching because of other providers' *pull* strategies, rather than *push* actions of current providers [41]. Underlying these incidents were patients' judgments of perceived service quality (“poor services compared to others”); benefits offered (“found a provider with better perks”), greater ease of access (“All of my specialists work for the same clinic and it's easier to make appointments and for all of the doctors to access my medical files”), and more highly‐skilled providers (“Better doctors”).

Incidents in this category included the pull of perceptions of better‐quality service (73.7%); better benefits or value (26.3%); perceptions that new providers were more skilled (7.9%); and perceptions that it was easier to access and coordinate services with the new provider (5.3%).

##### 3.1.2.3 Inconvenience

Inconvenience included CI where respondents switched providers because providers' services were inaccessible due to location, service hours, or available appointment times and long wait times during appointments. 12.8% of all voluntary switching incidents cited inconvenience‐related factors as one reason for switching.

Three subcategories of inconvenience‐related CIs emerged: location (63.4%) (“wanted a doctor closer to my house”), difficulty scheduling appointments (31.7%) (“difficult to get appointments”), and long wait times (7.3%) (“He keeps me waiting for long”).

##### 3.1.2.4 Pricing

Pricing included incidents where patients switched because of price‐related concerns, such as price increases, unfair pricing practices, or the inability to afford services. 12.5% of voluntary switching reported pricing as one reason for switching providers.

Pricing consisted of five subcategories: high prices, comparative price assessments, price increases, unaffordability, and unfair pricing practices. High prices (34.8%), where patients switched providers because they found the services too expensive, relative to some internal normative price (“They charged too much”). Comparative price assessments (19.6%) included respondents switching because they found the services costly compared to other providers (“Because I found a cheaper one”). Price increases, sub‐category (10.9%), where respondents switch providers because their provider raised prices (“I switched because they had gone up in price”). Unaffordability (21.7%) included patient switching because costs exceeded their financial capacity, often because of life event changes (“I lost my job and I needed to get something in my price range”), and not because services were overpriced per‐se. Unfair pricing practices (6.5%) included patients' switching because they perceived providers' pricing strategies were unjust, deceptive, or exploitative (“hidden fees”). In 10.9% of cases, respondents reported switching providers because of “price”, without elaborating further.

##### 3.1.2.5 Core Service Failures

For Core Service Failures (8.4% of all voluntary switching incidents), switching incidents involved mistakes or technical problems with the core service, either by omission or commission. Some respondents described these failures merely as a source of concern, while others reported severe consequences including unnecessary treatments, medical complications, and hospitalization.

Five subcategories of core service failures included: incorrect or missed diagnoses (38.4%) (“An appointment where symptoms were ignored and led to a hospitalization the next day”); negligence (26.9%) (“I was kept on medications longer than I should have been”); error in medical procedures (15.4%) (“They took 3 different occasions to draw my blood”); prescription drug errors (3.9%) (“They gave me incorrect medication dosage”); and administrative errors (11.5%) (“The PCP office forgot to call with my results & further advice. It took me 3 days & 12 phone calls to get someone on the phone to at least give me the results of my $500 test”). The remaining 3.9% incidents didn't elaborate on the “medical errors” to explain switching.

##### 3.1.2.6 Breakdown in Shared Decision‐Making

Our data identified breakdowns in shared decision‐making (SDM) as a CIC factor not previously reported in customer switching studies. In healthcare, SDM is a care‐delivery model where providers and patients collaborate on medical decisions about treatment. Physicians present options, and patients express their values and preferences [42, 43]. Ideally, both parties reach a consensus that aligns with the patient's values, resulting in benefits including improved treatment adherence, better disease coping, and improved health outcomes [44, 45, 46]. However, patients' emotional distress or limited health literacy can make it difficult to reach consensus. Additionally, patients' preferences for treatments that offer subjective value but no medical benefits (e.g., taking antibiotics for viral infections), the proliferation of often misleading online information, and providers' obligation to prevent harm and uphold care standards—guided by medical principles of beneficence and non‐maleficence [47, 48]—may hinder consensus. Together, these can result in breakdowns in shared decision‐making. In these cases, patients switched providers not due to core service failures (e.g., wrong test or medication, medical negligence) or functional failures in care delivery (e.g., poor interpersonal exchanges), but because of mismatches between their expectations or beliefs and those of their providers.

7.2% of all voluntary switching incidents indicated a breakdown in SDM as one reason for switching. These incidents reflected disparities in the volume or range of services expected (40.0%) (“the doctor was more about prescribing more meds, and I was looking for a doctor who was going to work to get me off some of the meds”); disparities in patient‐provider service philosophies (32.0%) (“I'm holistic and because he's plant‐based, I had hoped that he would be more unconventional”); and unmet expected outcomes (28.0%) (“she was not fixing me but just prescribing a lot of meds”).

##### 3.1.2.7 Perception of Service Environment

Patients' perceptions of the service environment emerged as another significant CIC, absent from existing customer switching research. This refers to the surroundings of the service encounter, including the ambiance (e.g., background noise, lighting, and temperature); design (e.g., color, furnishings, spatial layout); artifacts (e.g., signage and “wayfinding” artifacts) and social aspects (e.g., other customers, queues, crowding, and employee's physical characteristics) [49, 50].

CIs where respondents attributed switching to negative assessments of the service setting were included in the perceptions of the service environment category and accounted for 5.9% of all voluntary switching incidents.

Three subcategories emerged: physical setting (5.3%) (“stressful small space”); process design (26.3%) (“Changed to a more formal doctor's office as opposed to a clinic”); and social aspects (68.4%) including staff turnover issues (“The staff were constantly changing”), role assignment (“They started using PAs and I seldom if ever got to see the doctor”); crowding (“My appointments were always crowded”), and perceptions of employee characteristics (“I didn't like their beliefs, saw it in an article”).

##### 3.1.2.8 Other Incidents

Some respondents (8.4% of voluntary switching) reported switching for reasons other than those previously mentioned. These seldom‐mentioned incidents were labeled “Other.” Responses in this category included interest in trying new providers (“Nothing happened. I like trying new companies”), accidental switching because of misunderstandings (“I switched accidentally to another provider”), and seeking second opinions (“wanted a second opinion”).

### 3.2 Single and Multiple Factor Critical Incidents

To examine whether patients exhibit a low tolerance for certain CI or endure multiple types of incidents before they are motivated to switch providers, we investigated the proportion of patients who reported switching providers because of a single category of CIs (“Single factor incidents”) or more than one category of incidents (“Multiple factor incidents”). Most respondents (88.6%) switched because of single‐factor incidents, while others (11.4%) attributed switching to multiple‐factor incidents.

We also examined the distribution of the CIC as single or multiple incidents. As shown in Table 4, we found that some categories (e.g., pricing and attraction by competitors) often occurred as single‐incident factors, while others (e.g., core service failure and inconvenience) often occurred as multiple‐factor incidents. This suggests that some CIC, while exacerbating for patients, may not necessarily be reason enough to switch providers.

**TABLE 4:** Breakdown of single and multiple factor critical incidents among respondents.

Critical incident categories	Single factor incidents		Multiple factor incidents			
	One‐factor		2‐Factors		3‐Factors	
	*N*	%	*N*	%	*N*	%
Involuntary switching	286	97.3%	8	2.7%		
Service encounter failures	59	60.8%	34	35.1%	4	4.1%
Pricing	33	82.5%	7	17.5%		
Attraction by competitors	32	68.1%	13	27.7%	2	4.3%
Inconvenience	22	53.7%	18	43.9%	1	2.4%
Core service failures	11	40.7%	15	55.6%	1	3.7%
Breakdown in shared decision‐making	13	56.5%	8	34.8%	2	8.7%
Perception of the service environment	13	68.4%	6	31.6%		
Others	23	85.2%	4	14.8%		

## 4 Summary and Discussion

Our research investigates factors that motivate patients to switch providers from the patients' perspectives. We use the CIT, an inductive research method that relies on first‐hand patient reports to identify groups of grounded events associated with their switching behavior. Because CIT neither involves a priori determination of what is important to patients nor forces responses into any given framework, it reflects their normal thinking processes. Thus, our research makes several significant contributions to the literature.

First, our study complements and extends research on switching behavior in Health Services (HS) and service management (SM) literature. Regarding HS, our study reveals two reasons why patients switch providers, not previously identified in general models of customer switching: *breakdown in shared decision‐making* and *perception of the service environment*. By using CIT, we unpack service quality dimensions that motivate patients to switch providers voluntarily, thereby addressing a limitation of prior studies, which have relied on composite measures of service quality [21]. Thus, our work provides insights that can inform more targeted interventions to address patients switching providers.

Regarding SM research, we found that in sharp contrast to other services, many patients (> 50%) report switching for involuntary reasons, indicating that most provider switching does not occur because of patient dissatisfaction, but for reasons beyond their control. While healthcare organizations may have limited ability to address patient‐related involuntary CI, there is opportunity to mitigate provider‐ or organization‐related issues. For example, while provider organizations have little control over providers' departure, they can develop transition protocols to communicate [51] impending change and introduce new providers, transfer medical records [52], and manage clearly defined processes to support patients during transition. We also found that of the eight CIC mentioned from other service domains [53, 54], only six apply to healthcare. Together, these suggest that while findings from other domains may not fully transfer to healthcare, appropriating effective interventions from other services may be a reasonable strategy to curtail patient switching for the CI related to switching in healthcare that overlap with those in other industries.

Second, our finding that breakdowns in SDM and perception of the service environment contribute to provider switching highlights tensions within some popular healthcare delivery practices and the need for mutual adaptation between patients and providers. While patient‐centered care models emphasize patient autonomy and participation in SDM [42, 44], factors such as information asymmetry and patients' emotional distress can create challenges in reaching consensus. Furthermore, the proliferation of online health information can influence patients' perceptions and expectations in ways incongruent with providers' judgments. However, these differences can also reflect patients' legitimate values about their care requirements rather than unreasonable expectations. Providers, therefore, often face the challenge of balancing their professional obligations to offer evidence‐based care with the need to accommodate patient preferences, particularly in clinically ambiguous situations where multiple treatment approaches may be appropriate. Yet most patient‐centered care frameworks lack strategies to adequately manage this tension [55]. Our study underscores the need to equip providers with tools to navigate these tensions and reduce switching behavior.

The finding that negative perceptions of the service environment, particularly its social aspects, reveals deeper tensions in care‐delivery practices. For example, our research highlights tensions stemming from growing reliance on advanced practice practitioners such as physician assistants (PA)s to fulfill clinical responsibilities traditionally provided by physicians [56]. While this trend mitigates workforce shortages and ballooning healthcare costs [57], little is known about how patients perceive these changes. Our research found that some patients switch providers when they receive medical care from PAs (e.g., “They started using PAs and I seldom, if ever, got to see the doctor”), suggesting that an unintended consequence of using PAs may disrupt continuity of care and motivate provider switching. Thus, our work underscores the need to research patient preferences regarding which clinical services should be physician‐provided and not provided by PAs. Understanding these preferences could be critical to minimizing provider switching and care disruptions due to skill‐mix changes.

Another interesting finding on patients' perceptions of social aspects of the environment involves biases and prejudice against the provider (“I learned that my doctor was gay…I saw an lgbtq pin on his coat”). Although HS literature identifies problematic behaviors among patients, such as incivility, retaliation, and psychological victimization [58, 59], biases based on social identity traits, such as gender presentation and sexual orientation, have received less attention. We highlight the importance of more research in this area to provide a fuller understanding of current knowledge on bias in healthcare.

Overall, our work reveals that many patients voluntarily switch providers for legitimate reasons, such as medical and administrative errors or poor interpersonal exchanges with providers. While extant research tends to emphasize the downsides of patient switching behavior (e.g., discontinuity of care, poor health outcomes, etc.), our work underscores its important function in market regulation, redirecting demand from underperforming providers and incentivizing system‐wide improvements in care‐quality. Patients may also switch providers to redirect or escalate service experiences they perceive are not progressing satisfactorily [15]. It is important for policymakers and healthcare organizations to recognize the risks and opportunities inherent in switching when crafting strategies directed toward improving healthcare delivery.

Our work has limitations that can guide further research. First, we employed CIT, an exploratory approach that neither evaluates causal relationships nor validates identified constructs. Confirmatory research can extend our work by evaluating the relationships we uncover. Second, we relied on commercial online participant panels for our survey, which limited control over sample acquisition and prevented traditional assessments of nonresponse bias given limited insights into the overall size and demographics of the underlying user populations [60]. Moreover, recruitment challenges in our quota‐based sampling approach necessitated the relaxation of certain demographic quotas (e.g., race/ethnicity). Despite these limitations, we ultimately achieved a demographically diverse sample that was quota‐balanced on several demographic and socio‐economic characteristics to approximate national distributions. This helps mitigate, though not eliminate, generalizability concerns, and results should be interpreted within this context. Third, because customers with different attributes interpret and respond to events during service encounters in different ways, future research can analyze whether patients with different profiles (e.g., disease acuity) are more (or less) sensitive to service encounter aspects. Such research efforts could reveal distinct patient segments and provider switching typologies, enabling a better understanding of various patient groups.

## Conflicts of Interest

The authors declare no conflicts of interest.

## Data Availability

Research data are not shared.
